# Awareness of family physician residents of their roles in disaster health management: a cross-sectional study in Turkey

**DOI:** 10.1017/S146342362000047X

**Published:** 2020-10-28

**Authors:** Tarık Eren Yılmaz, Tuğba Yılmaz, Nüket Örnek Büken, Adem Özkara, Kerim Hakan Altıntaş

**Affiliations:** 1 Department of Family Medicine, Ankara Numune Training and Research Hospital, University of Health Sciences, Ankara, Turkey; 2 Department of History of Medicine and Medical Ethics, Faculty of Medicine, University of Hacettepe, Ankara, Turkey; 3 Department of Public Health, Faculty of Medicine, University of Hacettepe, Ankara, Turkey

**Keywords:** disaster ethics, disaster management, disaster medicine, family physician, pandemic ethics

## Abstract

**Aim:**

Family physicians are role models for their societies in disaster management and have an important place in it. This study was carried out during the specialty training of the residents, who are currently family physicians fighting against COVID-19 in the field, and was aimed to identify the awareness levels of residents regarding the roles and duties of family physicians before, during, and after disasters and to increase their awareness of disaster medicine and management.

**Background:**

The duties and responsibilities of a family physician in disasters should be a part of their specialty training. This study has contributed to the limited literature, increased awareness, and opened a new avenue of research for studies to be conducted with family physicians by demonstrating the current situation of family physicians in disaster management.

**Methods:**

This is an observational and descriptive study. The knowledge, experience, opinions, willingness, attitudes of the residents, and the awareness levels of the residents regarding their roles and duties in a disaster were evaluated along with their sociodemographic information. The surveys were applied in the family medicine clinics of the all residents by the interview method (*n* = 233).

**Findings:**

Only 9.2% of the residents stated that they had received training on disaster medicine where they currently work. The knowledge level of the residents on this subject was found as ‘Unsure’. In total, 80% of the residents stated that family physicians should have a role in disasters. It was found that 83.3% of the residents had never joined a disaster drill, 94.3% had never participated in making or applying a disaster plan, and 97.7% had never worked in any disaster.

**Conclusion:**

The residents participating in the study lacked not only information on disaster management but also experience. The residents’ willingness to receive training, work voluntarily, significantly question the curriculum, and specialize in disaster medicine were a positive outcome.

## Introduction

In the past, an approach based on crisis prevention was prevalent in disaster management and the most needed physician group was doctors and surgeons working in emergency services. However, risk management is at the center of the modern disaster management process and thus a new physician group is needed. Family physicians, who know the population at risk before a disaster strikes, can implement risk management on a regional scale and form an important health force in the field. Also in many countries the role of public health authorities and public health physicians is very important and usually they have the first responsibility in this area.

Family physicians will definitely take part in a number of tasks during and after a disaster, such as the first detection of the event, the collection and distribution of critical information, effective interventions by triage, and referral chain and rehabilitative activities. Roughly one-sixth of physicians, who are at the core of the overall group of health professionals, are family physicians serving in primary healthcare services, although this differs for each country (Republic of Turkey Ministry of Health, 2019). Therefore, family physicians and their residents need sufficient training for disasters to come even if they have not encountered one before. Family physicians are expected to be in contact with other units beforehand and to be ready for disasters by making a disaster plan (Physicians, 2010). This is also a requirement of disaster ethics. From an ethical perspective family physicians must work together and share information because they will need the support of each other. In these circumstances, despite the effective services available, family physicians will be dealing with most of the healthcare needs in the community due to the overwhelming number of critical health issues. They should make their medical decisions which must be ‘reasonable in the circumstances, based on the best evidence available at the time, made in accordance with government’s disaster plan, made as collaboratively as possible, designed to promote safe, and effective patient care’ (British Medical Association, 2020). If they make their decisions in this manner and in accordance with the circumstances, it will make it easier for them to overcome ethical dilemmas.

Furthermore, it may be reasonable for family physicians to have different ways of working in these circumstances. These may include; ‘a reduction or cancellation of non-essential services, a reduction or cancellation of home visits, widespread use of telephone triage, increased use of telephone and video consultation, greater use of email and messaging apps, the cancellation of all non-urgent appointments’ (British Medical Association, 2020).

### Important local and world issue; a new area for research

But there are not many publications available in the literature on the role of family physicians in disaster management despite the importance of this subject. The need for studies that will contribute to the literature on disaster health management in primary healthcare services is seen in our country and also all over the world.

The aim of this study is to determine the knowledge and awareness levels of family medicine residents who will serve in primary healthcare services and who need to be trained on this subject (Unfortunately, there is not a section on disaster health management in the standard curricula of family medicine residency training in Turkey). In line with this goal, it was planned to evaluate the knowledge, opinions, and experiences of residents on disasters along with their sociodemographic features, their awareness about their duties in a disaster, and their willingness to participate in disaster health management.

## Methods

### Study design

This is a cross-sectional, observational, and descriptive epidemiological survey study.

The surveys were administered to residents in Ankara under observation in the training halls of the clinics with which they were affiliated.

In order to reach sufficient participants between January and February 2015, relevant clinics were visited twice and multiple participation was prevented.

### Participants

The population of the study comprised the residents working in all family practice clinics in Ankara, the capital of Turkey, and 233 residents working in a total of 10 family practice clinics and centers were invited to participate in the study as the population. Efforts were made to reach the target population. A total of 183 people were reached by visiting all the clinics in Ankara, and 174 residents were included in the study after excluding 9 people who did not meet the study criteria (exclusion criteria: failure to complete the whole survey form, being in another residency program or city, refusal to give informed consent). Therefore, 74.7% of the total study population was reached.

### Measurement

A structured survey was prepared in accordance with the literature review carried out before the study and the job descriptions of family physicians during disasters as defined in ‘Application and Data Set Guide to Primary Healthcare Services’ by the Public Health Agency of Turkey, and it was made final after the pilot study.

The variables examined in the survey were age, gender, marital status, number of children, other people in the household, hospitals worked at, total working duration, total working duration in the current family practice clinic, institutions previously worked at, receiving disaster medicine training, participating in a drill, making a disaster plan, institutional membership related to disaster management, experience of a disaster by the person or a relative, willingness to train on disaster medicine, willingness to work voluntarily during disasters, knowing the job description in disasters, opinions on the necessity of family physicians’ roles in disasters, opinions on disaster medicine, and willingness to specialize in this subject.

### Ethical issues

Research approval was received from the Ankara Numune Training and Research Hospital Research Ethics Committee via E.14.339 registration number.

### Statistical analysis

Statistical evaluation was performed with SPSS 20 for Windows (IBM Corp., Armonk, NY, USA). The normal distribution of the numerical data was evaluated with the Kolmogorov–Smirnov test. Numerical variables were expressed as mean ± standard deviation and categorical variables as numbers and percentages. The Student *t*-test or Mann–Whitney *U* test was employed in comparisons of two groups for numerical variables while the two-way ANOVA test was employed in comparisons of three groups. Chi-square and Fisher exact chi-square tests were employed in the comparison of categorical data.

Under the heading of ‘Health Services in Emergencies and Disasters’, out of all the statements about job definitions before, during, and after a disaster, 14 of them were correct while 6 of them were false. For the correct statements, 1 point was given for the answer ‘No, this is not a duty of the physician’; 2 points were given for ‘I am unsure’; and 3 points were given for ‘Yes, this is true’. For false statements, reverse scoring was performed.

The scores that each individual received were summed and divided by the total number of statements. The mean knowledge level of each individual on disasters was thus obtained. Residents with mean knowledge levels below 1.5 were classified as having ‘insufficient’ knowledge levels, while residents with mean knowledge levels between 1.5 and 2.5 were classified as ‘unsure’ and residents with mean knowledge levels of 2.5 and above were classified as having ‘sufficient’ knowledge on disaster medicine. The Cronbach alpha coefficient was calculated and the *α* value was found to be 0.89 in order to measure the reliability of 20 statements prepared in this 3-point Likert-type scale, which measures the knowledge levels of disaster management. In statistical analyses, *P* < 0.05 was accepted as significant.

## Results

A total of 174 participants (118 women and 56 men) who completely answered the survey were included in the study. The mean age of the participants in the study was 29.2 ± 3.8 years. The median of time working as a physician was found as 29.5 months (range: 1–216), while the median of time working as a family medicine resident was 11 months (range: 1–42). Other sociodemographic information of the participants is shown in Table 1.

**Table 1.** Sociodemographic information of the family physician residents based on gender

Sociodemographic information	Whole population, *n* (%)	Male, *n* (%)	Female, *n* (%)
Age			
24–27	63 (36.2)	21 (37.5)	42 (35.6)
28–30	72 (41.4)	21 (37.5)	51 (43.2)
>30	39 (22.4)	14 (25.0)	25 (21.2)
Marital status			
Single	62 (35.6)	23 (41.1)	39 (33.1)
Divorced/Widowed	7 (4.1)	2 (3.5)	5 (4.1)
Married	105 (60.3)	31 (55.4)	74 (62.7)
Number of children			
0	127 (73)	41 (73.2)	86 (72.9)
1	37 (21.3)	11 (19.6)	26 (22)
2	10 (5.7)	4 (7.1)	6 (5.1)
Hospitals worked and trained at			
Ankara Numune RTH	61 (35.1)	16 (28.6)	45 (38.1)
Atatürk RTH	40 (23)	9 (16.1)	31 (26.3)
Ankara RTH	21 (12.1)	9 (16.1)	12 (10.2)
Ankara Dışkapı RTH	11 (6.3)	4 (7.1)	7 (5.9)
Ankara Keçiören RTH	2 (1.1)	0 (0)	2 (1.7)
Başkent UMF	15 (8.6)	5 (8.9)	10 (8.5)
GATA UMF	12 (6.9)	11 (19.6)	1 (0.8)
Ankara UMF	10 (5.7)	1 (1.8)	9 (7.6)
Hacettepe UMF	1 (0.6)	1 (1.8)	0 (0)
Turgut Özal UMF	1 (0.6)	0 (0)	1 (0.8)
Living with			
Spouse	59 (33.9)	15 (26.8)	44 (37.3)
Spouse and children	44 (25.3)	16 (28.6)	28 (23.7)
Alone	27 (15.5)	10 (17.9)	17 (14.4)
Parents	26 (14.9)	11 (19.6)	15 (12.7)
Other	18 (10.3)	4 (7.1)	14 (11.9)
Institutions previously worked at^a^			
Hospital Emergency Service	101 (58)	35 (62.5)	66 (55.9)
Community Health Center (CHC)	34 (19.5)	9 (16.1)	25 (21.2)
Family Health Center (FHC)	28 (16.1)	7 (12.5)	21 (17.8)
112 Emergency Health Station	21 (12.1)	9 (16.1)	12 (10.2)
Health Center	11 (6.3)	2 (3.6)	9 (7.6)
City Health Administrative (CHA)	10 (5.7)	2 (3.6)	8 (6.8)
112 Command and Control Center	10 (5.7)	2 (3.6)	8 (6.8)
Other	43 (24.7)	17 (30.4)	26 (22)

RTH = Research and Training Hospital; UMF = University Medical Faculty.

a Row percentages are given according to the population for this variable, where more than one option could be selected.

### Experiencing a disaster

Of the total participants, 33.9% (*n* = 59) stated that they had experienced a disaster. Fifty-four people had experienced an earthquake, while one had experienced a flood, and one a fire. The percentage of women who had experienced a disaster was higher than that of men (40.7% versus 19.6%; *P* = 0.006). These people were additionally asked whether they had suffered in any way and 27.1% (*n* = 16) of the 59 people who had experienced disasters, or 9.2% of the total participants, stated that they had suffered from the disaster they experienced. They were asked to state whether this suffering was physical, financial, or emotional. Twelve people stated that they were emotionally affected while two stated that they had financially suffered. None were physically injured. The other 115 participants had not experienced a disaster before. Additionally, 159 people answered the question ‘Have any of your relatives suffered from a disaster?’ negatively, while 15 people (8.6%) answered ‘Yes, they suffered’. All of those whose relatives had suffered a disaster were female (12.7%; *P* = 0.012).

### Disaster medicine training and willingness

Data on the training of residents in disaster medicine and disaster management and their willingness to receive training on this subject are given in Table 2.

**Table 2.** Distribution of family physician residents’ educational status and willingness to receive training on disaster medicine and disaster management by gender

Educational status and willingness	Whole Population, *n* (%)	Male, *n* (%)	Female, *n* (%)	*P*
Receiving Training on Disaster Medicine/Management in the Clinic				
No	145 (83.3)	44 (78.6)	101 (85.6)	0.500
Yes	16 (9.2)	7 (12.5)	9 (7.6)	
I do not remember	13 (7.5)	5 (8.9)	8 (6.8)	
Receiving Training on Disaster Medicine/Management at Any Point in Educational History (Before or After Graduation)				
No	130 (74.7)	41 (73.2)	89 (75.4)	0.469
Yes	27 (15.5)	11 (19.6)	16 (13.6)	
I do not remember	17 (9.8)	4 (7.1)	13 (11)	
Inclusion of Disaster Medicine/Management Lessons in Specialty Training				
No	104 (59.8)	36 (64.3)	68 (57.6)	0.354
Yes	2 (1.1)	0 (0)	2 (1.7)	
I do not remember	68 (39.1)	20 (35.7)	48 (40.7)	
Willingness to Train on Disaster Medicine/Management				
No	21	13	8	0.004*
Yes	153 (87.9)	43 (76.8)	110 (93.2)	
Request of Including Disaster Medicine/Management in Specialty Training Curriculum				
No	30 (17.2)	18 (32.1)	12 (10.2)	0.001*
Yes	144 (82.8)	38 (67.9)	106 (89.8)	

* *P* < 0.05, statistical significance.

Also the willingness to receive training again on disaster medicine of the residents was investigated in the group of residents who had received previous training. It was found that 88.9% (*n* = 24) of the residents who had received previous training on disaster medicine (*n* = 27; 15.5% of whole population), 87.7% (*n* = 114) of the residents who had not received previous training (*n* = 130; 74.7% of whole population), and 88.2% (*n* = 15) of the residents who did not remember receiving training (*n* = 17; 9.8% of whole population) wanted training on disaster medicine (*P* = 0.984). The percentage of residents who answered ‘yes’ to the question of whether they were harmed in a disaster was higher among the residents who wanted training on disaster medicine than in the group that did not (27.6% versus 0%; *P* = 0.001). It was also found that the residents who experienced a disaster, whether they suffered from it or not, were more willing to receive training than those who had not experienced a disaster (37.9% versus 4.8%; *P* = 0.002).

Examining how the suffering of the participants’ relatives in any disaster affected them, it was found that 50.0% of the residents who stated that ‘it did not affect them much’, 83.3% of the residents who stated that ‘it somewhat affected them’, and all of the residents who stated that ‘it deeply affected them’ wanted disaster medicine subjects to be thoroughly taught in family physician training (*P* = 0.033). It was further found that 74.1% (*n* = 20) of the residents who had received any training on disaster medicine so far and 84.6% (*n* = 110) of the residents who had not received any training wanted disaster medicine to be thoroughly taught in family physician training (*P* = 0.445).

### Participation in disaster drills and disaster plan preparation

The disaster drill experiences of the residents were investigated and 83.3% (*n* = 145) of the participants stated that they had not participated in any disaster drills before. The type and place of the drills were asked of those who had participated in them and it was seen that 10 people had participated in earthquake drills, 5 people in fire drills, and 8 people in general drills. On the other hand, 164 people had not participated in any disaster planning and were inexperienced in terms of risk management, which is one of the vital elements of disaster management (Table 3).

**Table 3.** Distribution of family physician residents’ experiences with disasters and disaster management, and opinions on disaster medicine by gender (*n* = 174)

Participating in disaster management and disaster medicine	Whole population, *n* (%)	Male, *n* (%)	Female, *n* (%)	*P*
Participated in a disaster drill	29 (16.7)	11 (19.6)	18 (15.3)	0.516
Participated in disaster planning	10 (5.7)	8 (14.3)	2 (1.7)	0.001*
Worked as a physician during a disaster	4 (2.3)	1 (1.8)	3 (2.5)	0.750
Voluntarily worked during a disaster	2 (50)	1 (100)	1 (33.3)	
Is a member of an institution fighting disasters	2** (1.1)	1 (1.8)	1 (0.8)	
Willing to voluntarily work if a disaster breaks out				
No	20 (11.5)	12 (21.4)	8 (6.8)	0.011*
Yes	108 (62.1)	34 (60.7)	74 (62.7)	
Unsure	46 (26.4)	10 (17.9)	36 (30.5)	
Can family physicians apply to a new sub-branch to be established with the focus of disaster medicine?				
Absolutely no	4 (2.3)	3 (5.4)	1 (0.8)	0.042*
No	15 (8.6)	6 (10.7)	9 (7.6)	
Unsure	33 (19)	11 (19.6)	22 (18.6)	
Yes	82 (47.1)	19 (33.9)	63 (53.4)	
Absolutely yes	40 (23)	17 (30.4)	23 (19.5)	
Willing to specialize in disaster medicine (*n* = 133)				
No	53 (39.8)	13 (35.1)	40 (41.7)	0.556
Yes	80 (60.2)	24 (64.9)	56 (58.3)	

* *P* < 0.05, statistical significance.

** NMRT = National Medical Rescue Team.

### Work experience and willingness to work as a physician during a disaster

The question of ‘Have you ever worked as a physician during a disaster before?’ was asked in order to learn about the participants’ experiences in the crisis management part of the disaster management process. In response, 170 people, or 97.7% of the participating residents, stated that they had not (Table 3).

The question of ‘Would you like to work as a volunteer if a disaster broke out today?’ was asked in order to learn about the participants’ willingness to take part in disaster management. According to the answers, this willingness was significantly high in both male and female residents (60.7% versus 62.7%) (*P* = 0.011) (Table 3). Examining how the suffering of their relatives in a disaster had affected the participants, 50% of the residents who had stated that ‘it did not affect much’, 83.3% of the residents who had stated that ‘it somewhat affected them’, and 85.7% of the residents who had stated that ‘it deeply affected them’ were willing to voluntarily work if a disaster broke out (*P* = 0.047).

The opinions of the residents on specializing in disaster medicine are also shown in Table 3. It was observed that none of the participants who stated that ‘it did not affect them much’ did not want to specialize in disaster medicine, while 66.7% of the residents who stated that ‘it somewhat affected them’ and 71.4% of the residents who stated that ‘it deeply affected them’ wanted to specialize in disaster medicine (*P* = 0.045).

### Role of family physicians in disaster and their knowledge levels

‘Should family physicians have a role in disasters?’ was asked in order to learn the participants’ opinions on the necessity of their roles in disasters. In the group that replied positively to this question, the percentage of female residents was higher (*P* = 0.023). Furthermore, it was found that those who answered ‘yes’ to this question also answered ‘yes’ to the question ‘Have you received any training on disaster medicine so far?’ more often compared to the groups that answered ‘no’ or ‘I am unsure’ (respectively 18.0%, 5.6%, and 5.9%; *P* = 0.042). Again, the percentage of residents answering ‘yes’ to this question was higher in the resident group that wanted ‘thorough teaching of disaster medicine issues in the family practice specialty training curriculum’ compared to the group that did not (88.2% versus 40.0%; *P* = 0.001). Similarly, the percentage of residents who believed that family physicians should have a role in approaching disasters was higher in the resident group that wanted to receive training on disaster medicine compared to the group that did not (86.3% versus 33.3%; *P* = 0.001). On the other hand, the percentage of residents who answered ‘yes’ to this question was higher in the group that wanted to specialize in disaster medicine compared to the group that did not (92.6% versus 81.1%; *P* = 0.032).

In short, it was found that female residents who have received training, are willing to train, and want to specialize in this field significantly support the view that ‘family physicians should have a role in disasters’.

The opinions of the residents showing their knowledge level scores on the job definitions of a family physician before, during, and after a disaster are shown in Table 4 based on percentages.

**Table 4.** Awareness of family physician residents of their job descriptions in disaster health management

Duties of family physicians in disaster health management	Whole population, *n* (%)		
	Not a duty	It is a duty	Unsure
Before a disaster			
Prepares disaster plan by conducting risk and resource analysis in the region	71 (40.8)	**53 (30.5)**	50 (28.7)
Plans to make first aid awareness for the public and conducts training activities in line with the plans made	30 (17.2)	**113 (64.9)**	31 (17.8)
Carries out activities to raise the awareness of the public about accidents and risky situations that may be encountered in daily life	23 (13.2)	**129 (74.1)**	22 (12.6)
Supplies and stores vaccines, medicines, and medical supplies	41 (23.6)	**106 (60.9)**	27 (15.5)
Conducts necessary public and personnel training within the framework of the disaster plan	42 (24.1)	**91 (52.3)**	41 (23.6)
Carries out drills within disaster management	76 (43.7)	**57 (32.8)**	41 (23.6)
During a disaster			
Implements the disaster plan and provides therapeutic services	34 (19.5)	**107 (61.5)**	33 (19)
Provides the necessary *logistics support* to the emergency health stations in the region during the disaster^a^	**54 (31)**	83 (47.7)	37 (21.3)
Provides acute medical intervention during the disaster	17 (9.8)	**140 (80.5)**	17 (9.8)
Performs *all administrative and religious procedures* related to the identification and burial of the dead in deaths caused by the disaster^a^	**115 (66.1)**	26 (14.9)	33 (19)
Performs *all kinds of medical interventions* for those affected by the disaster and keeps records^a^	**44 (25.3)**	91 (52.3)	39 (22.4)
Performs the emergency response and *transfers* patients to the appropriate medical institution if necessary^a^	**23 (13.2)**	135 (77.6)	16 (9.2)
If necessary, *leads the appropriate team* or provides consultancy^a^	**23 (13.2)**	128 (73.6)	23 (13.2)
*Transfers* disaster victims if necessary or leaves them in the region^a^	**24 (13.8)**	122 (70.1)	28 (16.1)
After a disaster			
Rehabilitates society after the disaster	13 (7.5)	**143 (82.2)**	18 (10.3)
Provides psychological support to society after the disaster	15 (8.6)	**138 (79.3)**	21 (12.1)
Follows the necessary psychological and other special measures such as shelter, nutrition, and education for children	47 (27)	**90 (51.7)**	37 (21.3)
Informs the population about the prevention of infectious diseases and ensures that personal measures are taken	6 (3.4)	**155 (89.1)**	13 (7.5)
Tries to provide the usual level of services in terms of vaccinations, chemoprophylaxis, and health education	12 (6.9)	**145 (83.3)**	17 (9.8)
Takes environmental safety measures such as those for water safety, food safety, vector control, waste control, etc.	76 (43.7)	**64 (36.8)**	34 (19.5)

Bold values show correct answers and percentages.

a False statements: those written in italics are false phrases.

According to the mean knowledge level calculated from the answers given to the true and false statements, the total mean knowledge level score of the residents was determined as 2.2 ± 0.4 or, in other words, ‘unsure’. The mean knowledge level score of the residents in the pre-disaster period was calculated as 2.3 ± 0.5 (‘unsure’); during the disaster period, it was calculated as 1.9 ± 0.3 (‘unsure’); and during the post-disaster period, it was 2.7 ± 0.4 (‘sufficient’).

Furthermore, a strong positive correlation was found between the knowledge levels of the pre-disaster and post-disaster periods (*r* = 0.613, *P* < 0.001), while a negative (inverse) correlation was found between the knowledge levels of the pre-disaster and disaster periods (*r* = −0.352, *P* < 0.001) and between the knowledge levels of the disaster and post-disaster periods (*r* = −0.407, *P* < 0.001).

The post-disaster knowledge level was found higher among female residents compared to male residents (2.6 ± 0.4 versus 2.4 ± 0.5; *P* = 0.003), while the knowledge levels for the periods before and during a disaster did not change based on gender.

Additionally, the mean knowledge level score was found higher among residents who answered the question ‘Should family physicians have a role in disasters?’ positively compared to others who answered ‘no’ or ‘unsure’ (2.7 ± 0.4 versus 2.2 ± 0.5 versus 2.4 ± 0.4; *P* = 0.004). The knowledge levels for the periods before, during, and after a disaster did not differ according to the other demographic findings and the answers given to the statements (*P* > 0.05). However, the total knowledge level scores of the residents who had previously worked in health centers, which are primary healthcare service institutions, were significantly higher than those who had worked in other institutions (2.1 versus 2.6; *P* = 0.045).

## Discussion

The fact that only 9.2% (*n* = 16) of all residents stated that they had received disaster training in their current family practice clinic shows that this issue is not emphasized enough in family practice clinics. It was found in another study carried out on this issue that most of the family physicians (73.8%) had not participated in a training session about disasters in the last two years (Pekez-Pavliško *et al.*, 2018). Furthermore, the few positive answers of the resident who had started their training recently to the question of whether they had received lessons on disaster medicine in their orientation training or training curricula (‘Yes, there is’: 1.1%, *n* = 2) suggests that this subject is considered unimportant. It was emphasized in the study of Uddin *et al.* (2008) that this problem exists in other branches as well. It was also stated in that study that there is not enough coverage of preparation for emergencies and disasters in the specialty training curricula in many branches including anesthesia, surgery, internal medicine, pediatrics, emergency medicine, and family practice, and also that there are no published reports on this subject. It was emphasized that some training modules should be prepared in order to solve this problem and these training modules must be adapted to the specialty training curricula of the branches (Uddin *et al.*, 2008).

The fact that only 15.5% (*n* = 27) of the participants had received lessons in their entire educational history is thought-provoking. This study thus shows that a training and education system on disaster preparation is necessary, especially in a country such as Turkey with a high disaster rate, given that the participants of this study had received their medical educations in different parts of the country. As mentioned before, it is seen that health professionals, who are the most important actors during disasters, and physicians, the most important among health professionals, are lacking in disaster management training. Similarly, the study of Sinha *et al.* (2008), carried out with medical students, showed that those students had little theoretical and practical knowledge on disaster preparation. Such an educational deficit will bring failure in disaster management in the future. It was demonstrated in other studies conducted in different countries in different parts of the world that proper training is not provided for disaster health management and that this is a general global problem (Martin *et al.*, 2006; Shealy *et al.*, 2006; Bagatell and Wiese, 2008; Uddin *et al.*, 2008; AlShowair *et al.*, 2018). This shows that disaster medicine lessons should be included not only in specialty training but also in the undergraduate education of medical students and these lessons should be standardized in the curricula.

It was emphasized in the study of Pekez-Pavliško *et al.* (2018) conducted with family physicians that all health professionals must be trained in preventing and preparing for emergencies and disasters and such training must be mandatory. Furthermore, 50.0% of the nurses participating in the study of Achora and Kamanyire (2016) stated that the issue of disaster preparation should be compulsory in undergraduate education and emphasized the need for and necessity of disaster management training for health professionals with different titles and from different branches. This is also valid for family physicians; in the study of Pekez-Pavliško *et al.* (2018), 50.0% of the participants thought that training for disaster recovery must be mandatory in the license renewal of family physicians.

It was found that residents were willing to train on this topic. In the study of Pekez-Pavliško *et al.* (2018), the positive statements of family physicians given by choosing the highest scores on disaster preparation and intervention requirements showed their willingness and positive attitudes on this subject in a similar manner.

The percentage of residents who had received any previous training on disaster medicine was found to be higher in the group that replied positively to the question ‘Should family physicians have a role in disasters?’ compared to the group that answered ‘No’ or ‘Unsure’, and this shows that training brings along a sense of responsibility. Furthermore, the mean knowledge level score for the post-disaster period was found higher among residents who had positively answered the question ‘Should family physicians have a role in disasters?’ compared to the residents who answered ‘No’ or ‘Unsure’, and this demonstrates that the knowledge levels of the residents who have a sense of responsibility and embrace health disaster management within their own discipline are higher. This led us to conclude that education, responsibility, and knowledge level affect each other in a positive manner. In general, many of the residents expressed their sense of responsibility and the awareness that they should have a role in disaster management, and this also shows that the vast majority of residents have a sense of duty in this regard.

The high percentage of residents who answered ‘yes’ to the question of whether family physicians should have a role in handling disasters may show that the residents who believe that family physicians should have roles in disaster management also want to specialize in disasters. Thus, it became apparent that those with a sense of duty wanted their training to be more sufficient. In other words, this study showed that residents who think that they have a role in disasters want to undergo thorough training in accordance with this role and that they want it to be provided in the course of their specialty training process.

Examining how the suffering of the participants’ relatives in any disaster affected them, it was found that all of the residents who stated that ‘it deeply affected them’ wanted disaster medicine subjects to be thoroughly taught in family physician training. This relationship suggests that the willingness of residents to receive disaster medicine training can change according to past experiences. Furthermore, the residents who stated that ‘it deeply affected them’ were willing to work voluntarily if a disaster broke out. This relationship shows that the suffering of a relative in a disaster can change the approach of residents to disasters. A similar relationship was found between anxiety and disaster preparation in a previous study (Blessman *et al.*, 2007). In another study, it was shown that there was a relationship between anxiety and disaster preparation and that this anxiety developed after a previously experienced disaster (Dooley *et al.*, 1992). As can be seen from these examples, experiencing a disaster before or having a relative who suffered from a disaster positively affects the approach of physicians to disaster preparation and actively working during a disaster.

While the low number of participants whose relatives were affected by a disaster constitutes a limitation for the above-mentioned hypothesis, the percentage of residents who suffered in any disaster was higher in the group of residents who wanted to receive training about disaster medicine than in the unwilling group, and this also supports the hypothesis. In other words, it can be said that personally suffering from a disaster can make physicians more sensitive in approaching disasters. The same result was found among all residents who had experienced a disaster. As stated in the study of AlShowair *et al.* (2018), the first intervention experience of most physicians in disasters usually occurs during a disaster that they experience themselves. This supports the idea that disaster response may not be known or the willingness to learn about it may not be present without first experiencing a disaster.

The percentage of residents experiencing any disaster was higher among the group that said that family physicians should have a role in the approach to disasters compared to the group that said they should not, which suggests that disaster survivors may have more disaster awareness.

The residents were given job definitions related to disaster management, including six before a disaster, eight during a disaster, and six after a disaster. The general mean score of residents for the pre-disaster period was 2.3 ± 0.5 (‘unsure’), while the score was 1.9 ± 0.3 (‘unsure’) for the disaster period and 2.7 ± 0.4 (‘sufficient’) for the post-disaster period. In a study conducted on the disaster intervention capacity of primary healthcare workers in China, in which their knowledge, attitudes, and behaviors were examined, the levels were not found to be sufficient in a similar manner (67.23 ± 10.61 out of 100), and it was emphasized that health administrators must regulate training sessions to make primary healthcare workers more sufficient in their responses to emergencies and disasters (Zhiheng *et al.*, 2012). As emphasized in another study, the sufficiency of family physicians, who have critical roles, must be increased in order for them to intervene in emergencies in a more competent manner (Zhiheng *et al.*, 2012). It was also stated in a related study that family physicians who carry out preventive activities based on preventive medicine should engage in mitigation, recovery, and treatment activities as a serious aid force from the first moments of disasters in the response capacity (AlShowair *et al.*, 2018). Family physicians’ positions as the first contact point, their extensive approach, and their ability to address a large population also make them stand out in this field.

In another study, it was stated that family physicians believe that they are not ready for the required emergency response system in the event of a national or local disaster (Pekez-Pavliško *et al.*, 2018). In another study, it was emphasized that the preparedness of family physicians for disasters is still uncertain (AlShowair *et al.*, 2018). Furthermore, it was stated that family physicians do not have sufficient knowledge and attitudes, and it is still unknown where family physicians should be positioned in disaster management (AlShowair *et al.*, 2018). These results show that similar setbacks exist in different parts of the world.

When the total working durations of the residents were grouped as one, two, three, or four years or more, a relationship was not found between the working duration and the knowledge level scores. Similarly, a difference was not found between the knowledge level scores of the groups when grouping the residents as first, second, and third year family medicine residents. Therefore, the working duration must be supported with sufficient training on the subject in order to increase the knowledge. It is apparent that in-service training programs are needed in disaster health management and disaster medicine. In the study of Pekez-Pavliško *et al.* (2018), it was found that working time, type of practice, and working environment did not affect the knowledge and attitude of family physicians about disaster management. However, the intervention capacities of more experienced physicians were found to be significantly higher in the study of Zhiheng *et al.* (2012) conducted among primary healthcare workers (*P* < 0.05). It was found in their multiple linear regression model that gender, title, type of work area, professional experience, and previous training on the subject were the main factors affecting the capacity for emergency intervention in healthcare (Zhiheng *et al.*, 2012).

This study showed that the experience of the residents in disasters was lacking along with the detected deficiencies in training and knowledge in accordance with the following statements of the participants: 83.3% of the residents had not participated in any disaster drills before; only 5.7% (*n* = 10) of the residents had participated in making or implementing a disaster plan at any point in their professional lives; 170 people, or in other words 97.7% of the participating residents, had not worked in a disaster as a physician before; only 1.1% of the participants are members of an institution relevant to disaster management; and 66.1% of the residents had not experienced a disaster before. Similar situations were demonstrated in many other studies and the lack of experience was emphasized (Uddin *et al.*, 2008; Zhiheng *et al.*, 2012; AlShowair *et al.*, 2018). For example, one study showed that 82.5% of family physicians had not participated in a disaster drill and only 16.3% of the participants had worked in a disaster (Pekez-Pavliško *et al.*, 2018). As is seen, knowledge should be put into practice, and a multidisciplinary approach should be followed during this process (Glow *et al.*, 2013). A general disaster awareness and culture should be formed within all disciplines.

All of the findings of this study have indicated that the participating residents lacked not only information on disaster management but also experience.

## Limitations

One of the limitations of this study was including only false statements in the job definitions for the disaster period. Participants being more careful about false statements and the implicit phrasing in the false statements may have caused the knowledge level scores for the disaster period to be low. Furthermore, conducting this study in a female-dominated population can be considered as a limitation in the general evaluation of the findings. Another limitation for the study is the possibility of a selection bias as the residents with an interest in disaster health management are keener to participate. Also, the results cannot be generalized as this study was conducted in a single province.

## Conclusion

This study detected the awareness levels of residents working in family medicine clinics regarding their roles and duties before, during, and after disasters. Accordingly, it was found that the training, knowledge levels, experiences, and practices of the residents in terms of disaster medicine and management were not sufficient in general.

On the other hand, the residents’ willingness to receive training, work voluntarily, significantly question the curriculum, and specialize in disaster medicine were positive outcomes. Furthermore, residents believe in the necessity of their roles in a disaster even though they do not know their job definitions, and this showed us that residents with a sense of duty are on the rise.

### The political, communal, and ethical aspects of this innovative study

This study has shown that for a resilient and less vulnerable society with a high capacity to cope with risks and disasters, family physicians with increased awareness should be encouraged.

‘Family Physicians and Families Ready for Disasters’ will be able to be undertaken with family physicians who can serve as guides and leaders for society with integrative approaches. Indeed, the current global disaster of COVID-19 has shown that family physicians will have many duties in fighting against pandemics, such as being the first contact point, following patients both individually and on a social scale, and working to recover from the biopsychosocial destruction and help societies rise up after crises.

The crucial guidance of family physicians on ethical issues is likely to arise when providing care and treatment during the disasters and pandemics. It is also vital that family physicians seek to ensure their own well-being and to support their colleagues in the disasters.

### The developmental aspect of this study

With this study, residents were provided the opportunity to discover their roles in disaster health management, which is the general name of activities to be carried out before, during, and after disasters. This study increased the awareness of residents in Ankara regarding disaster medicine as well as attracting the attention of professors in the visited clinics, thus emphasizing the importance of the subject. As a result of this study, a ‘Disaster Management’ lesson was included in the remote training program provided to approximately 21 500 family physicians in Turkey within the scope of ‘Continuous Professional Development Training of Family Physicians’, contributing to the instruction of all family physicians in Turkey for the first time on this subject (University of Yıldırım Beyazıt, 2015).

### The achievement aspect of this innovation

Nowadays, during the COVID-19 pandemic outbreak, the participants of this study also have been checking the well-being of all people who have come from abroad and the population in self-isolation by telemedicine and follow them within the primary healthcare services in Turkey as new family physicians in the primary health care. They also have been working with the filiation teams and with all other family physicians in Turkey. They have reached all the people (n:251.028) who have contacted with COVID-19 patients (n:65.111). After this method of screening the chain of contact in the infectious disease, these people were isolated from the others after they have been tested (Özgenç, 2020). In addition, in family health centers, which are the first point of contact, they have been making the preliminary diagnosis for undiagnosed patients, carry out their referrals, and contribute to the promotion of disaster awareness within the population that they are responsible for in terms of staying at home and ensuring personal hygiene. Mortality and attack rate of Covid-19 pandemic are considerably low in Turkey thanks to the diligence of the family physicians (Özgenç, 2020). This intensive study conducted on the role of the family physicians in Turkey’s success in terms of filiation and early treatment has great importance.
